# The quantifying relationship between the remission duration and the cardiovascular and kidney outcomes in the patients with primary nephrotic syndrome

**DOI:** 10.1080/0886022X.2022.2143377

**Published:** 2022-11-12

**Authors:** Xuan Lai, Zhao Cui, Hua Zhang, Yi-miao Zhang, Fang Wang, Xin Wang, Li-qiang Meng, Xu-yang Cheng, Gang Liu, Ming-hui Zhao

**Affiliations:** a Renal Division, Peking University First Hospital; Institute of Nephrology, Peking University; Key Laboratory of Renal Disease, Ministry of Health of China; Key Laboratory of CKD Prevention and Treatment, Ministry of Education of China, Beijing, China; b Geriatrics Department, Peking University Third Hospital, Beijing, China; c Research Center of Clinical Epidemiology, Peking University Third Hospital, Beijing, China; d Peking-Tsinghua Center for Life Sciences, Beijing, China

**Keywords:** Nephrotic syndrome, clinical remission, remission duration, arteriosclerotic cardiovascular disease, kidney outcome

## Abstract

**Background:**

Patients with persistent nephrotic-range proteinuria have a high risk of kidney dysfunction and cardiovascular events. Recently, the maintenance of proteinuria remission has been demonstrated to reduce the risk of kidney endpoint. However, the effect of remission duration on cardiovascular outcomes remains unclear.

**Methods:**

This study enrolled 982 patients with primary nephrotic syndrome who had achieved clinical remission. Remission duration was defined as the maintenance time (months) of the first remission. Arteriosclerotic cardiovascular disease (ASCVD) and kidney dysfunction (ESKD or eGFR reduction >50%) were the endpoints. Survival curves, Cox regression models, restricted cubic spline analysis were used and the cutoff time points were determined.

**Results:**

During the 38.3 months of follow-up, 161 (16.4%) patients developed ASCVD (51.3 per 1000 patient-years) and 52 (5.3%) patients developed kidney dysfunction (15.3 per 1000 patient-years). Multivariate analysis showed that remission duration was an independently protective factor to ASCVD, in which each one-year extension associated with a 15% reduction of the risk (HR, 0.854; 95% CI, 0.776 ∼ 0.940, *p* = .001). The initial time point was seven months for remission to present the protective effect to ASCVD and the maximum time point was 36 months. Remission duration was also an independently protective factor to kidney dysfunction. This effect was shown from the beginning of remission and reached the maximum at 26 months.

**Conclusions:**

The maintenance of proteinuria remission was crucial for the improvement of cardiovascular and kidney outcomes in nephrotic syndrome patients.

## Introduction

Nephrotic syndrome (NS) is characterized clinically by the presence of peripheral edema, heavy proteinuria, hypoalbuminemia, and hypercholesterolemia. Most adult patients with NS have primary glomerular diseases, including membranous nephropathy (MN), minimal change disease (MCD), and focal segmental glomerulosclerosis (FSGS) [1,2].

Patients with persistent high-grade nephrotic-range proteinuria have a high rate of deterioration to end stage kidney disease (ESKD) [3]. In contrast, complete remission (CR) is associated with excellent long-term kidney survival [4]. A relapse after remission is common in NS and impedes the long-term benefits of remission [5]. Studies have shown that benefits accrue in kidney survival proportionate to the amount of time with lower levels of proteinuria. Cattran et al. [6] examined the association of remission status at fixed landmarks (3, 6, 12, 24, and 36 months) after the date of first remission and the primary end point as ESKD or eGFR >50% reduction in MN patients. They found that maintaining remission associated with significantly reduced risk of the primary outcome at all landmarks. Similar associations between the duration of proteinuria remission and clinical outcome have also been observed in IgA nephropathy [7].

Little population-based data exist about the adults with primary NS and the risks of cardiovascular outcomes. In a cohort of 907 adults with nephrologist confirmed primary NS attributed to MCD, FSGS and MN, the adjusted rates of acute coronary syndrome, heart failure, ischemic stroke, venous thromboembolism, and death were significantly higher than those in 89,593 matched adults [8]. It suggests that clinical remission and long-term maintenance may reduce the risk of arteriosclerotic cardiovascular disease (ASCVD) in NS patients. However, quantifying the effect of remission duration on cardiovascular outcomes remains unclear.

Major controversies in the management of NS include the timing of immunosuppressive therapy. When only the hard end points of ASCVD and kidney dysfunction are used, evidence is largely lacking. However, most clinical trials use the surrogate outcome measured by the change in proteinuria. The time duration of maintaining clinical remission for the benefits of kidney and cardiovascular outcomes remains a major concern of physicians, patients, and health agencies. This study enrolled 982 primary NS patients who had achieved clinical remission, examined the quantitative relationship between the remission duration and the hard end points of ASCVD and kidney dysfunction, in order to provide reference for the usage of proteinuria remission as surrogate outcomes in the management of NS.

## Materials and methods

### Study population

A total of 982 consecutive adult patients with NS were enrolled in this study from a prospective follow-up cohort of Peking University First Hospital from 2007 to 2019 (Supplementary Figure 1). They all achieved clinical remission and were treated and followed up for more than 6 months.

The study protocol (number: 2014 [749]) was approved by the Ethics Committee of Peking University First Hospital and was in compliance with the purpose of the Declaration of Helsinki. All participants provided written informed consent before participating in the study.

### Definition of remission duration

According to KDIGO 2021 clinical practice guideline, treatment response of NS includes CR, partial remission (PR), and relapse [1]. CR is defined as proteinuria <0.3 g/d with a stable eGFR, PR is defined as a ≥ 50% reduction of proteinuria to <3.5 g/d with stable eGFR, and relapse is defined as an increase of proteinuria to >3.5 g/d after achieving clinical remission.

Remission duration is defined as the maintenance time (months) of the first remission. All the patients had achieved clinical remission (CR or PR) at the beginning of the observation period. Their remission duration was calculated from then on. When some patients got relapse, their remission duration was stopped. If the patients had maintained remission during the whole period, their remission duration was calculated until the last visit.

### Definitions of covariates

Smoking was defined as currently smoking or any history of smoking. Hypertension was defined as a previous diagnosis of hypertension, systolic blood pressure ≥140 mmHg or diastolic blood pressure ≥90 mmHg, or the current use of antihypertensive drugs. Mean arterial pressure (MAP)=diastolic blood pressure + 1/3 × (systolic blood pressure − diastolic blood pressure). Diabetes mellitus was defined as fasting plasma glucose ≥7.0 mmol/L, a self-reported history of diabetes mellitus, or current use of antidiabetic medication. Body mass index (BMI) was calculated with the following formula: BMI = weight (kg)/height^2^ (m^2^).

Infections included the local or systemic inflammatory reactions caused by pathogens, such as pulmonary infection, urinary system infection, digestive system infection, and so on. Thromboembolism included deep venous thrombosis, pulmonary embolism, renal venous thrombosis, and so on. Acute kidney injury was defined as an increase in serum creatinine > 0.3 mg/dL or >50% from the baseline within 48 h.

### Clinical outcomes and follow-up

The primary cardiovascular endpoint was the occurrence of ASCVD, which includes acute coronary syndrome (including myocardial infarction, stable or unstable angina, or coronary or other arterial revascularization), stroke, transient ischemic attack, or peripheral artery disease (including aortic aneurysm and all atherosclerotic sickness) [9].

The primary kidney endpoint was the occurrence of kidney dysfunction, which was a composite of ESKD [estimated glomerular filtration rate (eGFR) < 15 mL/min/1.73m^2^, dialysis, or kidney transplantation] or a reduction of eGFR > 50% from the baseline value at the time of enrollment. eGFR was calculated according to the Chronic Kidney Disease Epidemiology Collaboration equation [10].

### Statistical analysis

Continuous variables with normal distribution were expressed as means ± SDs, and variables with non-normal distribution were expressed as medians and interquartile ranges (IQR). Categorical variables were expressed as frequencies and proportions. According to distributions, independent sample *t* test or Mann–Whitney U test was used to compare differences between two groups for continuous variables, and chi-square test or Fisher exact test was used to compare differences between two groups for categorical variables. Univariate and multivariate linear regression analyses were performed to analyze the potential determinants of remission duration. Variables with significance in univariate analysis were included in multivariate analysis.

The incidence rate of ASCVD or kidney dysfunction was represented by the number of cases per 1000 patient-years. The maximum selection test was used to calculate the landmark time point. The survival curve was calculated by Kaplan–Meier method. Log-rank test was used to compare the incidence rates between the patients divided according to the landmark.

A univariate Cox proportional hazards regression model was used to investigate the factors associated with clinical outcomes. A multivariable Cox proportional hazards regression model was used to investigate associations between remission duration and outcomes. The remission duration was included into the Cox model as a continuous variable or categorical variable. When as a categorical variable, the group with remission duration lower than the landmark was used as reference. Three models were constructed to adjust the factors associated with remission duration. 24 h urinary protein was adjusted together with other baseline factors in model 1, remission status (CR or PR) was adjusted in model 2, and relapse in model 3. HR and 95% confidence interval (CI) were reported. The curve of HR over remission duration was drawn by restrictive cubic spline analysis, and piecewise regression was carried out to determine the initial cutoff point and stable cutoff point of HR decline.

Data were analyzed using SPSS Statistics version 24.0 (IBM) and R 4.0.5 software. A two-sided *p* < .05 was considered statistically significant.

## Results

### Clinical parameters of the study population

The baseline features of 982 patients were shown in Supplementary Table 1. The mean age was 49.8 ± 16.3 years, with a gender ratio of male/female as 1.4. 302 (30.8%) patients had a history of smoking, 536 (54.6%) patients had hypertension, and 220 (22.4%) patients had diabetes mellitus.

All patients received kidney biopsy. 746 (76.0%) patients were diagnosed as primary MN, 163 (16.6%) patients were MCD, and 53 (5.4%) patients had primary FSGS. At the time of remission, urinary protein was 2.0 (1.0, 2.7) g/24h, serum albumin was 34.4 ± 5.6 g/L, and eGFR was 87.1 ± 25.0 mL/min/1.73 m^2^. In terms of NS complications, 303 (30.9%) patients underwent infections, 45 (4.6%) patients had thromboembolisms, and 65 (6.6%) patients had acute kidney injury.

The time from kidney biopsy to clinical remission was 4.4 (1.4, 10.9) months; 461 (46.9%) patients achieved CR and 521 (53.1%) patients got PR. During the follow-up of 38.3 (22.2, 57.3) months, 445 (45.3%) patients underwent relapse.

### Factors associated with remission duration

The time of remission duration was positively associated with serum albumin, hemoglobin, eGFR, remission status (CR or PR), whereas it was negatively associated with gender, 24 h urinary protein at baseline, uric acid, immunosuppressive drugs and relapse in univariate analysis. After multivariable adjustment, remission duration remained significantly associated with 24 h urinary protein at baseline, remission status and relapse (Supplementary Table 2).

### Remission duration and the risk of ASCVD

During the median follow-up of 38.3 (22.2, 57.3) months, 161 (16.4%) patients developed ASCVD, with an incidence of 51.3 per 1000 patient-years. Cox regression analysis showed that the time of remission duration was independently associated with a 15% decreased risk of ASCVD (HR, 0.854; 95% CI, 0.776–0.940, *p* = .001) for each one-year extension of remission, after adjustment of baseline factors (Table 1). In the subgroup of MN patients (*n* = 746), the duration of remission maintenance was also an independently protective factor for ASCVD, with a 12% decreased risk of ASCVD for one-year longer duration (HR, 0.882; 95% CI, 0.794–0.979, *p* = .019) (Supplementary Table 3). Besides remission duration, higher eGFR was an independently protective factor for ASCVD as well. Older age, diabetes mellitus, LDL-C, and thromboembolism were independently risk factors for ASCVD. The protective effect of remission duration remained statistically significant after further adjustments for the related parameters, 24 h urinary protein at baseline, remission status, and relapse (Table 2).

**Table 1.** The risk factors of ASCVD in the patients with nephrotic syndrome after getting clinical remission (Cox regression).

	Univariate analysis		Multivariate analysis	
	HR (95% CI)	*p*	HR (95% CI)	*p*
Age (increased by one year)	1.076 (1.062–1.090)	<.001	1.057 (1.040–1.075)	<.001
Gender (male)	0.998 (0.731–1.363)	.990		
Smoking	2.211 (1.622–3.015)	<.001	1.324 (0.954–1.838)	.094
Hypertension	3.069 (2.133–4.417)	<.001	1.323 (0.892–1.963)	.164
Diabetes mellitus	2.814 (2.055–3.854)	<.001	1.420 (1.016–1.984)	.040
Serum albumin (increased by 1 g/L)	0.964 (0.939–0.991)	.009	1.002 (0.970–1.036)	.904
eGFR (increased by 1 ml/min/1.73m^2^)	0.972 (0.967–0.978)	<.001	0.988 (0.979–0.996)	.005
Uric acid (increased by 1 μmol/L)	1.002 (1.000–1.003)	.011	1.000 (0.998–1.001)	.710
Hemoglobin (increased by 1 g/L)	0.980 (0.972–0.989)	<.001	0.999 (0.989–1.001)	.912
LDL-C (increased by 1 mmol/L)	1.163 (1.053–1.284)	.003	1.203 (1.072–1.349)	.002
MAP (increased by 1 mmHg)	1.020 (1.005–1.035)	.011	0.993 (0.976–1.010)	.433
Kidney pathology (MCD vs. non–MCD)	0.592 (0.367–0.955)	.032	0.500 (0.290–0.864)	.013
ACEI/ARBs	1.564 (0.887–2.759)	.122		
Immunosuppressive therapies	1.785 (1.138–2.799)	.012	1.402 (0.868–2.266)	.167
Time from biopsy to remission (increased by one year)	0.931 (0.817–1.061)	.283		
Remission duration (increased by one year)	0.895 (0.822–0.975)	.011	0.854 (0.776–0.940)	.001
Infections	1.611 (1.179–2.201)	.003	1.075 (0.763–1.516)	.678
Thromboembolism	3.009 (1.842–4.914)	<.001	1.855 (1.093–3.148)	.022
Acute kidney injury	1.684 (1.018–2.783)	.042	0.860 (0.493–1.502)	.597

*Note:* ASCVD: arteriosclerotic cardiovascular disease; eGFR: estimated glomerular filtration rate; LDL-C: low-density lipoprotein cholesterol; MAP: mean arterial pressure; MCD: minimal change disease; non-MCD: membranous nephropathy, focal segmental glomerulosclerosis, and others; ACEI: angiotensin converting enzyme inhibitor; ARB: angiotensin receptor blocker; HR: hazard ratio; CI: confidence interval.

**Table 2.** The prognostic role of remission duration to ASCVD with adjustment of related parameters (Cox regression).

	Model 1		Model 2		Model 3	
	HR (95% CI)	*p*	HR (95% CI)	*p*	HR (95% CI)	*p*
Remission duration	0.858 (0.780–0.944)	.002	0.853 (0.768–0.947)	.003	0.825 (0.739–0.921)	.001
24 h urinary protein at baseline	1.119 (0.926–1.353)	.245				
Remission status (CR or PR)			1.013 (0.690–1.489)	.946		
Relapse					0.772 (0.527–1.130)	.183

*Note:* ASCVD: arteriosclerotic cardiovascular disease; HR: hazard ratio; CI: confidence interval.

**Figure 1.**
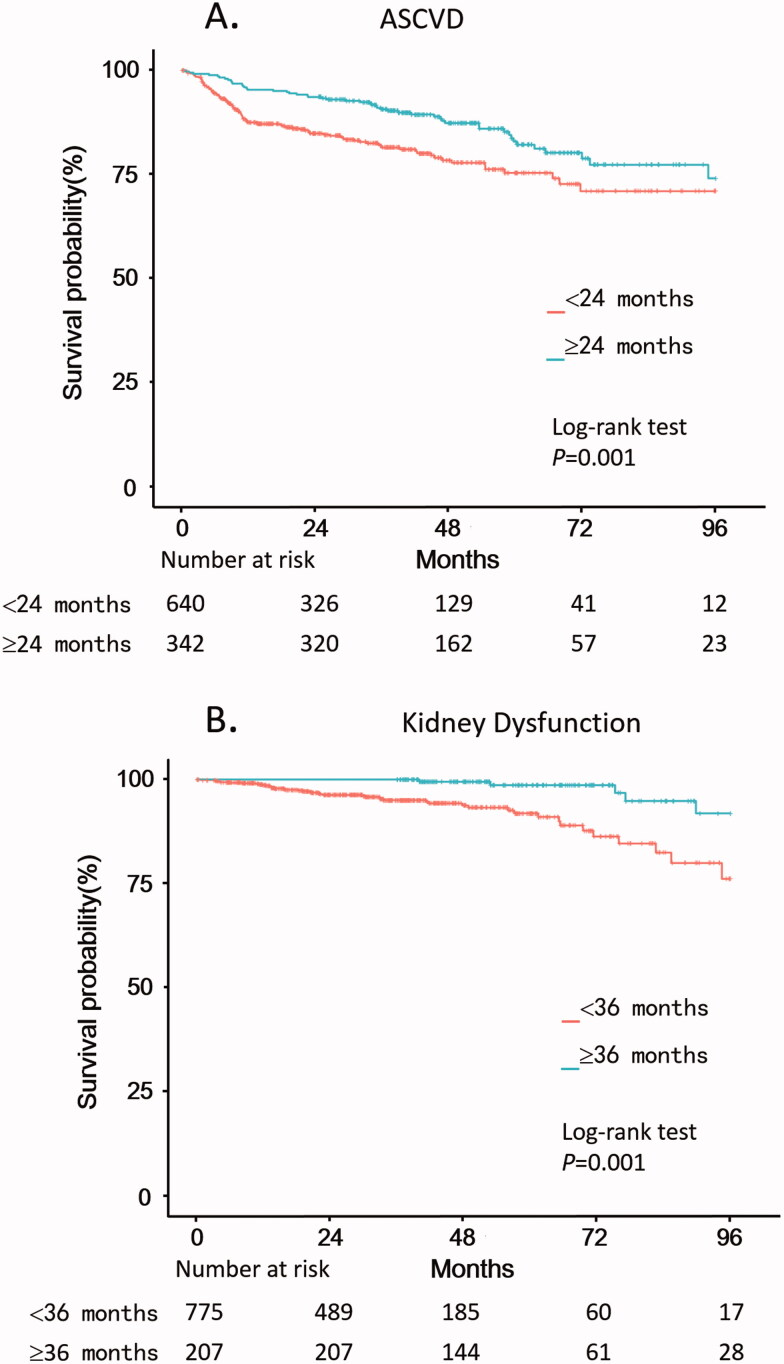
Kaplan–Meier survival curves of ASCVD (A) with NS patients divided by the landmark of remission duration (24 months), and Kaplan–Meier survival curves of kidney dysfunction (B) with NS patients divided by the landmark of remission duration (36 months). NS: nephrotic syndrome; ASCVD: arteriosclerotic cardiovascular disease.

The landmark of remission duration for the endpoint of ASCVD was calculated as 24 months by the maximum selection test, and the patients were divided into two groups accordingly (Supplementary Table 4). In the patients with remission duration < 24 months, the incidence of ASCVD was 66.4 per 1000 patient-years. While in the others the incidence was 34.1 per 1000 patient-years. The difference between the two groups was statistically significant (log-rank test, *p* = .001) (Figure 1(A)). After adjustment of baseline factors, remission duration ≥24 months was an independently protective factor to ASCVD (HR, 0.469; 95% CI, 0.330–0.667, *p* < .001) (Supplementary Table 5). The results remained statistically significant after further adjustments for 24 h urinary protein, remission status, and relapse (Supplementary Table 6).

### Remission duration and the risk of kidney dysfunction

During the follow-up, 52 (5.3%) patients underwent kidney dysfunction, with an incidence of 15.3 per 1000 patient-years. Cox regression analysis showed that remission duration was independently associated with a 30% decreased risk of kidney dysfunction (HR, 0.703; 95% CI, 0.585–0.845, *p* < .001) for each one-year extension of remission, after adjustment of baseline factors (Table 3). In the subgroup of MN patients (*n* = 746), the duration of remission maintenance was also an independently protective factor for kidney outcome, with a 25% decreased risk of kidney dysfunction for one-year longer duration (HR, 0.748; 95% CI, 0.625–0.896, *p* = .002) (Supplementary Table 7). Besides remission duration, higher eGFR was an independently protective factor for kidney dysfunction as well. Hypertension, the time from kidney biopsy to clinical remission, and thromboembolism were independently risk factors for kidney dysfunction. The prognostic effect of remission duration remained statistically significant after further adjustments for the related parameters, 24 h urinary protein at baseline, remission status, and relapse (Table 4).

**Table 3.** The risk factors of kidney dysfunction in the patients with nephrotic syndrome after getting clinical remission (Cox regression).

	Univariate analysis		Multivariate analysis	
	HR (95% CI)	*p*	HR (95% CI)	*p*
Age (increased by one year)	1.055 (1.033–1.077)	<.001	1.026 (1.001–1.051)	.039
Gender (male)	0.998 (0.576–1.727)	.993		
Smoking	1.897 (1.097–3.280)	.022	1.151 (0.641–2.068)	.637
Hypertension	5.628 (2.538–12.481)	<.001	2.874 (1.239–6.667)	.014
Diabetes mellitus	1.768 (0.989–3.161)	.055		
Serum albumin (increased by 1 g/L)	0.975 (0.930–1.022)	.294		
eGFR (increased by 1 ml/min/1.73m^2^)	0.967 (0.957–0.977)	<.001	0.983 (0.968–0.997)	.019
Uric acid (increased by 1 μmol/L)	1.003 (1.001–1.006)	.004	1.000 (0.997–1.003)	.867
Hemoglobin (increased by 1 g/L)	0.977 (0.963–0.992)	.003	0.999 (0.983–1.016)	.952
LDL-C (increased by 1 mmol/L)	0.936 (0.741–1.183)	.581		
MAP (increased by 1 mmHg)	1.032 (1.006–1.060)	.017	0.997 (0.970–1.024)	.811
Kidney pathology (MCD vs. non-MCD)	0.184 (0.045–0.756)	.019	0.099 (0.013–0.758)	.026
ACEI/ARBs	1.070 (0.424–2.699)	.886		
Immunosuppressive therapies	2.474 (0.983–6.224)	.054		
Time from biopsy to remission (increased by one year)	1.192 (1.017–1.397)	.030	1.221 (1.038–1.437)	.016
Remission duration (increased by one year)	0.775 (0.668–0.899)	.001	0.703 (0.585–0.845)	<.001
Infections	1.478 (0.853–2.560)	.163		
Thromboembolism	5.816 (2.882–11.736)	<.001	3.468 (1.641–7.333)	.001
Acute kidney injury	4.573 (2.395–8.731)	<.001	2.053 (1.001–4.213)	.008

*Note:* eGFR: estimated glomerular filtration rate; LDL-C: low-density lipoprotein cholesterol; MAP: mean arterial pressure; MCD: minimal change disease; non-MCD: membranous nephropathy, focal segmental glomerulosclerosis, and others; ACEI: angiotensin converting enzyme inhibitor; ARB: angiotensin receptor blocker; HR: hazard ratio; CI: confidence interval.

**Table 4.** The prognostic role of remission duration to kidney dysfunction with adjustment of related parameters (Cox regression).

	Model 1		Model 2		Model 3	
	HR (95% CI)	*p*	HR (95% CI)	*p*	HR (95% CI)	*p*
Remission duration	0.708 (0.588–0.852)	<.001	0.756 (0.628–0.910)	.003	0.718 (0.588–0.876)	.001
24 h urinary protein at baseline	1.156 (0.816–1.637)	.414				
Remission status (CR or PR)			0.393 (0.161–0.956)	.039		
Relapse					1.226 (0.562–2.675)	.609

*Note:* HR: hazard ratio; CI: confidence interval.

The landmark of remission duration for the endpoint of kidney dysfunction was calculated as 36 months by the maximum selection test, and the patients were divided into two groups accordingly (Supplementary Table 4). In the patients with remission duration <36 months, the incidence of kidney dysfunction was 19.2 per 1000 patient-years, while in the counterpart group it was 7.2 per 1000 patient-years (log-rank test, *p* = .001) (Figure 1(B)). After adjustment of baseline factors, remission duration ≥36 months was an independently protective factor to kidney dysfunction (HR, 0.183; 95% CI, 0.072–0.467, *p* < .001) (Supplementary Table 8). The results remained statistically significant after further adjustments for 24 h urinary protein at baseline, remission status, and relapse (Supplementary Table 9).

### Hazard ratio curve over remission duration

**Figure 2.**
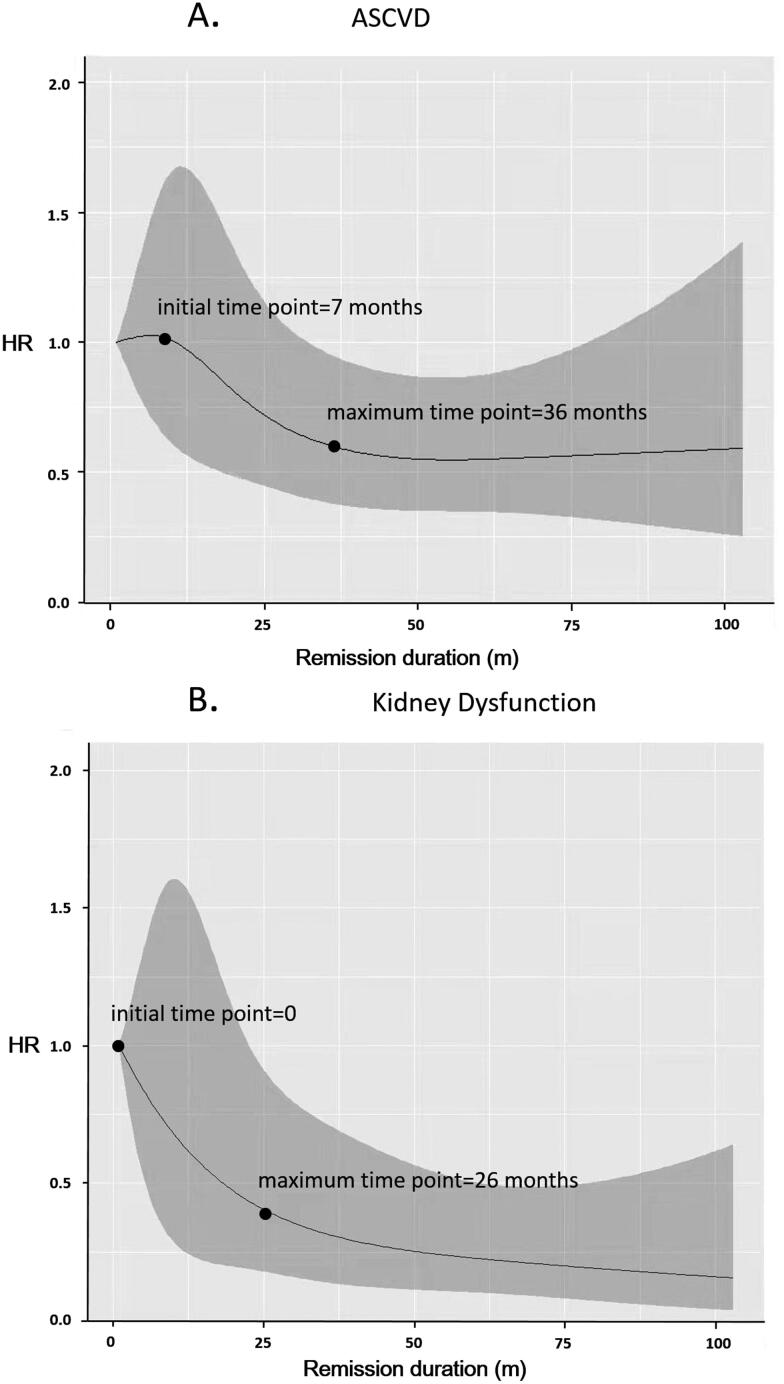
The curve of HR to ASCVD (A) and kidney dysfunction (B) over remission duration. ASCVD: arteriosclerotic cardiovascular disease.

The curve of HR to ASCVD over remission duration was drawn by restrictive cubic spline analysis, by taking HR as the vertical coordinate and remission duration as the horizontal coordinate (Figure 2(A)). The piecewise regression was carried out to determine the initial point and the stable point of HR decline. At the time of just achieving remission (duration = 0), no significant decrease of HR to ASCVD was shown. After seven months of maintaining remission (first cutoff point), the HR fell below 1.0, which showed the protective effect of remission to the risk of ASCVD. After that, along with the increase of remission duration, the HR to ASCVD was decreased rapidly. When the time reached 36 months (second cutoff point), the HR became stable and the extension of remission did not associate with further decrease of HR to ASCVD.

The curve of HR to kidney dysfunction over remission duration was presented in Figure 2(B). At the beginning of clinical remission, the HR to kidney dysfunction began to decrease rapidly and was below 1.0, which showed the immediately protective effect to the kidney outcomes once clinical remission was achieved. When the remission duration reached 26 months (cutoff point), the HR to kidney dysfunction gradually leveled off, and the extension of remission did not associate with further decrease of HR to kidney dysfunction.

## Discussion

In the present study, we determined the quantifying relationship between the surrogate outcome of NS as proteinuria remission and the hard outcomes of ASCVD and kidney dysfunction, using a large cohort of adult primary NS under regular longitudinal care within a tertiary hospital. The results showed that remission duration was an independently protective factor to the endpoint of CVD and CKD. For the benefits of cardiovascular outcomes, the remission duration needed to be maintained at least seven months. While for the benefits of kidney outcomes, the remission played the role once it was achieved. This is, for our best knowledge, the first study to define the specific time points for proteinuria remission to exert and to perform the stable protective effect to the endpoint of ASCVD in NS. These findings provide a reference for using the surrogate outcome of proteinuria remission in the management of NS, for the improvement of hard outcomes of cardiovascular and kidney prognosis.

In the current study, we observed an incidence of 51.3 per 1000 patient-years of ASCVD in NS patients, which is remarkably higher than that in general population, 6.6 per 1000 patient-years [11]. Recently, Go et al. [8] reported that the NS adults had higher adjusted rates of multiple types of cardiovascular outcomes and all-cause death compared with a large cohort of matched adults without diabetes, diagnosed NS, or known evidence of nephrotic-range proteinuria. An earlier analysis by Ordonez et al. [12] observed significantly higher adjusted relative risk (5.5) of myocardial infarction in NS patients. Mahmoodi et al. [13] investigated the risk of myocardial infarction, unstable angina, and ischemic stroke in 298 Dutch adults with NS, and reported an annual incidence of arterial thromboembolic events of 1.48%, with myocardial infarction and unstable angina much more common than peripheral artery disease or ischemic stroke. All these findings support the need to design more effective care strategies for NS patients who are at high risk of ASCVD.

We found that the risk of ASCVD could be decreased 15% by one-year extension of remission duration. This effect remained significant no matter which remission status, PR or CR, was achieved. At the initiation of proteinuria remission, no significant decrease of HR was observed, suggesting that the remission duration did not show protective effect on ASCVD. The cutoff time point for HR to fall down below 1.0 was seven months, suggesting that the remission duration should be maintained for seven months or longer, so that to reduce the risk of ASCVD. Then, the remission performed continuous and incremental protective effect to ASCVD. We found the landmark of remission duration as 24 months that the risk of ASCVD was reduced 54% in the patients achieving it. When the time of remission reached 36 months, the HR became stable, which suggests the protective effect approaching the maximum, and the extension of remission duration did not show further protective effect to ASCVD.

All the above results provided strong evidence that remission duration was an independently protective factor for ASCVD in NS patients, and for the first time clarified the definite time points for the initiation, landmark, and maximum of this protective effect. Although the literature supports a benefit to ASCVD by proteinuria remission [11,14–17], there was a lack of findings on the relationship between remission duration and ASCVD in NS. We examined this gap in evidence by looking at long-term outcomes of NS patients who achieved at least PR. We measured the durability of remission on outcomes using the landmark analysis method [18]. This method is commonly used in cancer studies to assess drug efficacy, and recently was applied in the analysis of the remitting/relapsing course of MN [6]. By this analysis we provided a more accessible result about the time-dependent benefit to ASCVD by the duration of proteinuria remission, which is much practical for the physicians to decide therapeutic strategies according to the risks of outcomes.

The possible mechanism underlying the relationship between proteinuria and ASCVD may be the endothelial dysfunction and inflammation [19]. Deckert et al. [20] proposed that the protein leakage into urine is a reflection of extensive vascular injury, suggesting systemic vascular endothelial dysfunction, possibly accompanied by hemodynamic disturbances or endothelial matrix dysfunction, which may increase the infiltration of lipoproteins from the blood into the vessel wall. Proteinuria may also act with hyperphosphatemia to increase the risk of ASCVD [19–21], which involve the NaPi-IIa, fibroblast growth factor 23, and klotho [22,23].

It has been fully demonstrated that proteinuria is an important risk factor for deterioration of kidney dysfunction, and the massive proteinuria in NS significantly increase the risk of ESKD [1]. Unlike the initiation time point of seven months for the benefit of ASCVD, the risk of kidney dysfunction was reduced once the proteinuria remission was achieved. It is consistent with the findings from Cattran et al. [6]. They found that MN patients with remission durations as short as 3 months had improved renal prognosis compared with the patients who relapsed. Separate analyses for PR and CR yielded similar results. This highlighted the necessity and value of achieving proteinuria remission in favor of the kidney outcome, set aside the unexpected relapse. The cumulative time effect of remission associated with greater improvement of kidney survival. We found that for every one-year increase in remission duration, the risk of kidney dysfunction decreased 30%. At the landmark of three years, the risk of kidney endpoints was reduced 82%. These findings validate the importance of maintaining remission, PR or CR, for the long-term outcomes in NS.

## Conclusion

In conclusion, this study demonstrated that remission duration was an independent protective factor for cardiovascular and kidney outcomes in the patients with NS. The time points of initiation and landmark of remission duration for the benefits of prognosis could be used as a reference time in the clinical practice for NS patients.

## Significant statement

Patients with persistent nephrotic-range proteinuria have a high risk of kidney dysfunction and cardiovascular events. However, the effect of the time of maintaining clinical remission on cardiovascular outcomes remains unclear. The results of this present study showed that remission duration was an independent protective factor for the endpoint of arteriosclerotic cardiovascular disease (ASCVD) and kidney dysfunction in primary nephrotic syndrome (NS) patients. This is, for our best knowledge, the first study to define the specific time points for clinical remission to exert and to perform the stable protective effect to the endpoint of ASCVD in primary NS patients. These findings provide a reference for maintaining remission in the clinical practice of NS, for the improvement of cardiovascular and kidney prognosis.

## Supplementary Material

Supplemental Material

## Data Availability

The data underlying this article are available in the article and in its online supplementary material.
