# Supplementary material for: The quantifying relationship between the remission duration and the cardiovascular and kidney outcomes in the patients with primary nephrotic syndrome
Source: Ren Fail. 2022 Nov 12;44(1):1915–23. doi: 10.1080/0886022X.2022.2143377 (PMC9662000; doi:10.1080/0886022X.2022.2143377)
Supplement: Supplemental Material [file IRNF_A_2143377_SM9772.pdf]

Supplementary Table 1. Clinical characteristics of the study population at the time of clinical remission.

Variables	N=982
Age, y	49.8±16.3
Sex, male/female	571/411
Body mass index, kg/m^2	24.6±3.5
Smoking, n (%)	302 (30.8)
Hypertension, n (%)	536 (54.6)
Diabetes mellitus, n (%)	220 (22.4)
Kidney pathology, n (%)	
Membranous nephropathy	746 (76.0)
Minimal change disease	163 (16.6)
Focal segmental glomerulosclerosis	53 (5.4)
Others	20 (2.0)
Urinary protein, g/24h	2.0 (1.0-2.7)
Serum albumin, g/L	34.4±5.6
Hematuria, n (%)	586 (59.7)
Serum creatinine, µmol/L	87.0±35.6
eGFR, ml/min/1.73m^2	87.1±25.0
Hemoglobin, g/L	137.0±18.2
Uric acid, mmol/L	365.8±100.3
Total cholesterol, mmol/L	5.8±1.9
Triglycerides, mmol/L	1.9 (1.3-2.7)
HDL-C, mmol/L	1.5±0.6
LDL-C, mmol/L	3.2±1.3
Treatments	
ACEI/ARBs, n (%)	856 (87.2)
Immunosuppressive therapies, n (%)	767 (78.1)
Corticosteroids alone, n (%)	124 (12.6)
Cyclophosphamide + corticosteroids, n (%)	333 (33.9)
Calcineurin inhibitor w/o corticosteroids, n (%)	486 (49.5)
Rituximab, n (%)	34 (3.5)
Mycophenolate mofetil, n (%)	147 (15.0)
Leflunomide, n (%)	158 (16.1)
Clinical remission	
Complete remission, n (%)	461 (46.9)
Partial remission, n (%)	521 (53.1)
Remission duration, m	14.0 (5.2-32.3)
Relapse, n (%)	445 (45.3)
Infections, n (%)	303 (30.9)
Thromboembolisms, n (%)	45 (4.6)
Acute kidney injury, n (%)	65 (6.6)
Follow-up duration, m	38.3 (22.2-57.3)

ESKD, n (%)	33 (3.4)
eGFR reduction >50% from baseline, n (%)	19 (1.9)
ASCVD, n (%)	161 (16.4)

eGFR, estimated glomerular filtration rate; HDL-C, high-density lipoprotein cholesterol; LDL-C, low-density lipoprotein cholesterol; ACEI, angiotensin converting enzyme inhibitor; ARB, angiotensin receptor blocker; ESKD, end stage kidney disease; ASCVD, arteriosclerotic cardiovascular disease. Continuous and normally distributed variables were presented as mean ± SD; continuous and non-normally distributed variables were presented as median, IQR; categorical variables were presented as number (%).

Supplementary Table 2. Parameters related to remission duration (linear regression).

Variables	Univariate		Multivariate	
	B	***P***	***B***	***P***
Age	0.003	0. 452		
Sex	-0.272	0.022	-0.137	0.276
Body mass index	-0.015	0.394		
Smoking	-0.085	0.504		
Hypertension	-0.157	0.183		
Diabetes mellitus	0.048	0.732		
Urinary protein	-0.176	0.002	0.143	0.015
Serum albumin	0.029	0.006	0.010	0.343
eGFR	0.006	0.016	0.002	0.319
Uric acid	-0.002	<0.001	-0.001	0.190
Hemoglobin	0.007	0.042	0.005	0.098
Total cholesterol	-0.029	0.346		
LDL-C	-0.079	0.069		
Mean arterial pressure	-0.010	0.094		
ACEI/ARBs	0.135	0.443		
Immunosuppressive drugs	-0.344	0.015	-0.040	0.765
NS duration	0.057	0.177		
Remission status (CR or PR)	1.390	<0.001	0.990	<0.001
Relapse	-1.363	<0.001	-0.851	<0.001
Remission after first treatment	0.166	0.305		
Infections	0.110	0.388		
Thromboembolisms	-0.348	0.215		
Acute kidney injury	-0.028	0.907		

eGFR, estimated glomerular filtration rate; LDL-C, low-density lipoprotein cholesterol; ACEI, angiotensin converting enzyme inhibitor; ARB, angiotensin receptor blocker.

Supplementary Table 3. The risk factors of ASCVD in the patients with MN (n=746) after getting clinical remission (Cox regression).

Variables	Univariate analysis		Multivariate analysis	
	**HR (95% CI)**	***P***	**HR (95% CI)**	***P***
Age (increased by 1 year)	1.081 (1.065-1.098)	<0.001	1.060 (1.040-1.080)	<0.001
Gender (male)	1.034 (0.727-1.470)	0.853		
Smoking	1.930 (1.363-2.732)	<0.001	1.289 (0.891-1.864)	0.178
Hypertension	2.485 (1.659-3.724)	<0.001	1.207 (0.789-1.845)	0.386
Diabetes mellitus	2.500 (1.758-3.554)	<0.001	1.300 (0.895-1.888)	0.168
Serum albumin (increased by 1 g/L)	0.977 (0.945-1.010)	0.173		
eGFR (increased by 1 ml/min/1.73m^2)	0.968 (0.961-0.975)	<0.001	0.987 (0.978-0.997)	0.010
Uric acid (increased by 1 µmol/L)	1.001 (1.000-1.003)	0.108		
Hemoglobin (increased by 1 g/L)	0.985 (0.975-0.994)	0.002	1.003 (0.992-1.013)	0.642
LDL-C (increased by 1 mmol/L)	1.226 (1.086-1.384)	0.001	1.214 (1.080-1.364)	0.001
MAP (increased by 1 mmHg)	1.014 (0.998-1.031)	0.095		
ACEI/ARBs	0.825 (0.403-1.690)	0.599		
Immunosuppressive therapies	2.150 (1.320-3.500)	0.002	1.523 (0.912-2.543)	0.108
Time from kidney biopsy to clinical remission (increased by 1 year)	0.976 (0.853-1.117)	0.727		
Remission duration (increased by 1 year)	0.895 (0.815-0.990)	0.031	0.882 (0.794-0.979)	0.019
Infections	1.689 (1.187-2.402)	0.004	1.065 (0.725-1.564)	0.750
Thromboembolism	2.718 (1.531-4.824)	0.001	1.509 (0.823-2.767)	0.183
Acute kidney injury	1.706 (0.796-3.656)	0.170		

ASCVD, arteriosclerotic cardiovascular disease; eGFR, estimated glomerular filtration rate; LDL-C, low-density lipoprotein cholesterol; MAP, mean arterial pressure; ACEI, angiotensin converting enzyme inhibitor; ARB, angiotensin receptor blocker; HR, hazard ratio; CI, confidence interval.

Supplementary Table 4. The clinical characteristics of patients stratified by the landmarks of remission duration.

Variables	Remission duration					
	< 24 m (n=640)	≥ 24 m (n=342)	*P*	< 36 m (n=774)	≥ 36 m (n=208)	*P*
Age, y	49.5±16.1	50.2±16.7	0.571	49.4±16.3	51.0±16.5	0.227
Sex, male/female	389/251	182/160	0.022	463/312	108/99	0.050
Body mass index, kg/m^2	24.6±3.5	24.7±3.4	0.547	24.6±3.5	24.6±3.4	0.807
Smoking, n (%)	204 (31.9)	98 (28.7)	0.298	240 (31.0)	62 (30.0)	0.778
Hypertension, n (%)	359 (56.1)	177 (51.8)	0.193	427 (55.1)	109 (52.7)	0.531
Diabetes mellitus, n (%)	141 (22.0)	79 (23.1)	0.702	170 (21.9)	50 (24.2)	0.496
Kidney pathology, n (%)			0.013			0.007
Membranous nephropathy	504 (78.8)	242 (70.8)		604 (78.0)	142 (68.3)	
Minimal change disease	97 (15.2)	66 (19.3)		122 (15.8)	41 (19.7)	
Focal segmental glomerulosclerosis	31 (4.8)	22 (6.4)		38 (4.9)	15 (7.2)	
Others	8 (1.3)	12 (3.5)		11 (1.4)	9 (4.3)	
Urinary protein, g/24h	2.0 (1.0-2.8)	1.8 (0.7-2.6)	0.014	2.0 (1.0-2.7)	1.8 (0.7-2.6)	0.079
Serum albumin, g/L	34.0±5.4	35.3±5.8	0.001	34.3±5.5	35.0±5.7	0.075
Serum creatinine, µmol/L	87.9±40.9	85.4±22.9	0.304	87.4±38.2	85.5±23.6	0.497
eGFR, ml/min/1.73m^2	86.4±24.7	88.6±25.5	0.190	86.9±25.0	88.0±25.2	0.571
Hemoglobin, g/L	136.4±18.7	138.1±17.1	0.172	136.7±18.4	138.3±17.5	0.266
Uric acid, mmol/L	373.0±103.5	352.3±92.7	0.002	369.8±100.7	350.6±97.3	0.015
Glucose, mmol/L	5.6±1.3	5.6±1.4	0.492	5.6±1.4	5.5±1.1	0.170
Total cholesterol, mmol/L	5.9±1.9	5.7±1.9	0.145	5.9±1.9	5.8±1.8	0.437
Triglycerides, mmol/L	1.9 (1.3-2.7)	1.9 (1.3-2.6)	0.252	1.9 (1.3-2.7)	1.8 (1.3-2.5)	0.227
HDL-C, mmol/L	1.5±0.6	1.6±0.6	0.679	1.5±0.6	1.6±0.6	0.475
LDL-C, mmol/L	3.3±1.4	3.1±1.3	0.048	3.2±1.4	3.1±1.3	0.191

Mean arterial pressure, mmHg	89.5±10.2	88.9±9.4	0.389	89.5±10.0	88.6±9.3	0.246
Treatments						
ACEI/ARBs, n (%)	560 (87.5)	296 (86.5)	0.671	678 (87.5)	178 (86.0)	0.568
Immunosuppressive therapies, n (%)	511 (79.8)	256 (74.9)	0.072	616 (79.5)	151 (72.9)	0.043
Treatment responses						
Remission status			<0.001			<0.001
Complete remission, n (%)	227 (35.5)	234 (68.4)		309 (39.9)	152 (73.4)	
Partial remission, n (%)	413 (64.5)	108 (31.6)		466 (60.1)	55 (26.6)	
Remission after first treatment, n (%)	536 (83.8)	293 (85.7)	0.429	653 (84.3)	176 (85.0)	0.787
Remission duration, m	7.0 (3.5-13.3)	38.5 (32.0-53.0)	<0.001	9.6 (4.0-20.5)	49.6 (39.6-65.4)	<0.001
Relapse, n (%)	367 (57.3)	78 (22.8)	<0.001	404 (52.1)	41 (19.8)	<0.001
Infections, n (%)	195 (30.5)	108 (31.6)	0.720	238 (30.7)	65 (31.4)	0.848
Thromboembolisms, n (%)	34 (5.3)	11 (3.2)	0.135	37 (4.8)	8 (3.9)	0.578
Acute kidney injury, n (%)	44 (6.9)	21 (6.1)	0.659	50 (6.5)	15 (7.2)	0.683
Time from kidney biopsy to clinical remission, m	4.2 (1.3-9.6)	4.8 (1.6-15.3)	0.023	4.2 (1.4-10.3)	5.0 (1.5-14.0)	0.233
Kidney dysfunction, n (%)	38 (5.9)	14 (4.1)	0.219	44 (5.7)	8 (3.9)	0.301
ASCVD, n (%)	111 (17.3)	50 (14.6)	0.272	126 (16.3)	35 (16.9)	0.822

eGFR, estimated glomerular filtration rate; HDL-C, high-density lipoprotein cholesterol; LDL-C, low-density lipoprotein cholesterol; ACEI, angiotensin converting enzyme inhibitor; ARB, angiotensin receptor blocker; ASCVD, arteriosclerotic cardiovascular disease. Continuous and normally distributed variables were presented as mean ± SD; continuous and non-normally distributed variables were presented as median, IQR; categorical variables were presented as number (%).

Supplementary Table 5. The risk factors of ASCVD in patients with nephrotic syndrome after getting clinical remission (Cox regression, remission duration as categorical variable).

Variables	Univariate analysis		Multivariate analysis	
	HR (95% CI)	*P*	HR (95% CI)	*P*
Age (increased by 1 year)	1.076 (1.062-1.090)	<0.001	1.058 (1.040-1.075)	<0.001
Gender (male)	0.998 (0.731-1.363)	0.990		
Smoking	2.211 (1.622-3.015)	<0.001	1.362 (0.981-1.890)	0.065
Hypertension	3.069 (2.133-4.417)	<0.001	1.305 (0.879-1.938)	0.187
Diabetes mellitus	2.814 (2.055-3.854)	<0.001	1.457 (1.042-2.038)	0.028
Serum albumin (increased by 1 g/L)	0.964 (0.939-0.991)	0.009	1.002 (0.970-1.035)	0.904
eGFR (increased by 1 ml/min/1.73m2)	0.972 (0.967-0.978)	<0.001	0.987 (0.979-0.996)	0.003
Uric acid (increased by 1 μmol/L)	1.002 (1.000-1.003)	0.011	1.000 (0.998-1.001)	0.636
Hemoglobin (increased by 1 g/L)	0.980 (0.972-0.989)	<0.001	0.999 (0.989-1.009)	0.888
LDL-C (increased by 1 mmol/L)	1.163 (1.053-1.284)	0.003	1.211 (1.079-1.358)	0.001
MAP (increased by 1 mmHg)	1.020 (1.005-1.035)	0.011	0.994 (0.977-1.011)	0.462
Kidney pathology (MCD vs. non-MCD)	0.592 (0.367-0.955)	0.032	0.484 (0.280-0.837)	0.009
ACEI/ARBs	1.564 (0.887-2.759)	0.122		
Immunosuppressive therapies	1.785 (1.138-2.799)	0.012	1.391 (0.861-2.248)	0.177
Time from kidney biopsy to clinical remission (increased by 1 year)	0.931 (0.817-1.061)	0.283		
Remission duration (≥24 vs. <24 months)	0.571 (0.408-0.801)	0.001	0.469 (0.330-0.667)	<0.001
Infections	1.611 (1.179-2.201)	0.003	1.064 (0.754-1.500)	0.725
Thromboembolism	3.009 (1.842-4.914)	<0.001	1.882 (1.111-3.187)	0.019
Acute kidney injury	1.684 (1.018-2.783)	0.042	0.832 (0.475-1.454)	0.518

ASCVD, arteriosclerotic cardiovascular disease; eGFR, estimated glomerular filtration rate; LDL-C, low-density lipoprotein cholesterol; MAP, mean arterial pressure; MCD, minimal change disease; non-MCD, membranous nephropathy, focal segmental glomerulosclerosis, and others; ACEI, angiotensin converting enzyme inhibitor; ARB, angiotensin receptor blocker; HR, hazard ratio; CI, confidence interval.

Supplementary Table 6. The prognostic role of remission duration to ASCVD with adjustment of related parameters (Cox regression, remission duration as categorical variable).

Variables	Model 1		Model 2		Model 3	
	HR (95% CI)	*P*	HR (95% CI)	*P*	HR (95% CI)	*P*
Remission duration (≥24 vs. <24 months)	0.479 (0.336-0.683)	<0.001	0.467 (0.323-0.675)	<0.001	0.415 (0.282-0.611)	<0.001
24h urinary protein at baseline	1.083 (0.893-1.314)	0.418				
Remission status (CR or PR)			1.017 (0.704-1.469)	0.927		
relapse					0.752 (0.521-1.084)	0.127

ASCVD, arteriosclerotic cardiovascular disease; HR, hazard ratio; CI, confidence interval.

Supplementary Table 7. The risk factors of kidney dysfunction in the patients with MN (n=746) after getting clinical remission (Cox regression).

**Variables**	**Univariate analysis**		**Multivariate analysis**	
	**HR (95% CI)**	***P***	**HR (95% CI)**	***P***
Age (increased by 1 year)	1.071 (1.044-1.100)	<0.001	1.047 (1.015-1.079)	0.004
Gender (male)	0.947 (0.518-1.732)	0.860		
Smoking	1.556 (0.849-2.852)	0.153		
Hypertension	3.742 (1.664-8.415)	0.001	2.111 (0.914-4.874)	0.080
Diabetes mellitus	1.839 (0.987-3.427)	0.055		
Serum albumin (increased by 1 g/L)	0.973 (0.919-1.031)	0.353		
eGFR (increased by 1 ml/min/1.73m^2)	0.967 (0.954-0.980)	<0.001	0.991 (0.975-1.007)	0.257
Uric acid (increased by 1 μmol/L)	1.003 (1.000-1.005)	0.059		
Hemoglobin (increased by 1 g/L)	0.982 (0.966-0.999)	0.039	1.001 (0.983-1.019)	0.944
LDL-C (increased by 1 mmol/L)	1.038 (0.808-1.334)	0.771		
MAP (increased by 1 mmHg)	1.026 (0.998-1.056)	0.068		
ACEI/ARBs	0.524 (0.185-1.482)	0.223		
Immunosuppressive therapies	3.296 (1.177-9.230)	0.023	1.789 (0.624-5.126)	0.279
Time from kidney biopsy to clinical remission (increased by 1 year)	1.251 (1.062-1.474)	0.007	1.265 (1.080-1.481)	0.004
Remission duration (increased by 1 year)	0.782 (0.665-0.919)	0.003	0.748 (0.625-0.896)	0.002
Infections	1.447 (0.786-2.664)	0.236		
Thromboembolism	7.867 (3.805-16.265)	<0.001	3.826 (1.738-8.423)	0.001
Acute kidney injury	9.370 (4.442-19.765)	<0.001	2.917 (1.313-6.483)	0.009

eGFR, estimated glomerular filtration rate; LDL-C, low-density lipoprotein cholesterol; MAP, mean arterial pressure; ACEI, angiotensin converting enzyme inhibitor; ARB, angiotensin receptor blocker; HR, hazard ratio; CI, confidence interval.

Supplementary Table 8. The risk factors of kidney dysfunction in patients with nephrotic syndrome after getting clinical remission (Cox regression, remission duration as categorical variable).

Variables	Univariate analysis		Multivariate analysis	
	HR (95% CI)	*P*	HR (95% CI)	*P*
Age (increased by 1 year)	1.055 (1.033-1.077)	<0.001	1.026 (1.001-1.051)	0.040
Gender (male)	0.998 (0.576-1.727)	0.993		
Smoking	1.897 (1.097-3.280)	0.022	1.230 (0.685-2.210)	0.488
Hypertension	5.628 (2.538-12.481)	<0.001	2.945 (1.271-6.826)	0.012
Diabetes mellitus	1.768 (0.989-3.161)	0.055		
Serum albumin (increased by 1 g/L)	0.975 (0.930-1.022)	0.294		
eGFR (increased by 1 ml/min/1.73m^2)	0.967 (0.957-0.977)	<0.001	0.983 (0.968-0.997)	0.018
Uric acid (increased by 1 μmol/L)	1.003 (1.001-1.006)	0.004	1.000 (0.997-1.003)	0.941
Hemoglobin (increased by 1 g/L)	0.977 (0.963-0.992)	0.003	0.999 (0.982-1.016)	0.899
LDL-C (increased by 1 mmol/L)	0.936 (0.741-1.183)	0.581		
MAP (increased by 1 mmHg)	1.032 (1.006-1.060)	0.017	0.996 (0.968-1.024)	0.759
Kidney pathology (MCD vs. non-MCD)	0.184 (0.045-0.756)	0.019	0.085 (0.011-0.653)	0.018
ACEI/ARBs	1.070 (0.424-2.699)	0.886		
Immunosuppressive therapies	2.474 (0.983-6.224)	0.054		
Time from kidney biopsy to clinical remission (increased by 1 year)	1.192 (1.017-1.397)	0.030	1.171 (0.999-1.373)	0.051
Remission duration (≥36 vs. <36 months)	0.289 (0.134-0.625)	0.002	0.183 (0.072-0.467)	<0.001
Infections	1.478 (0.853-2.560)	0.163		
Thromboembolism	5.816 (2.882-11.736)	<0.001	3.491 (1.659-7.344)	0.001
Acute kidney injury	4.573 (2.395-8.731)	<0.001	2.672 (1.304-5.477)	0.007

eGFR, estimated glomerular filtration rate; LDL-C, low-density lipoprotein cholesterol; MAP, mean arterial pressure; MCD, minimal change disease; non-MCD, membranous nephropathy, focal segmental glomerulosclerosis, and others; ACEI, angiotensin converting enzyme inhibitor; ARB, angiotensin receptor blocker; HR, hazard ratio; CI, confidence interval.

Supplementary Table 9. The prognostic role of remission duration to kidney dysfunction with adjustment of related parameters (Cox regression, remission duration as categorical variable).

Variables	Model 1		Model 2		Model 3	
	HR (95% CI)	*P*	HR (95% CI)	*P*	HR (95% CI)	*P*
Remission duration (≥36 vs. <36 months)	0.186 (0.073-0.473)	<0.001	0.228 (0.089-0.588)	0.002	0.211 (0.079-0.561)	0.002
24h urinary protein at baseline	1.232 (0.874-1.736)	0.234				
Remission status (CR or PR)			0.330 (0.138-0.793)	0.013		
relapse					1.430 (0.675-3.030)	0.350

HR, hazard ratio; CI, confidence interval.

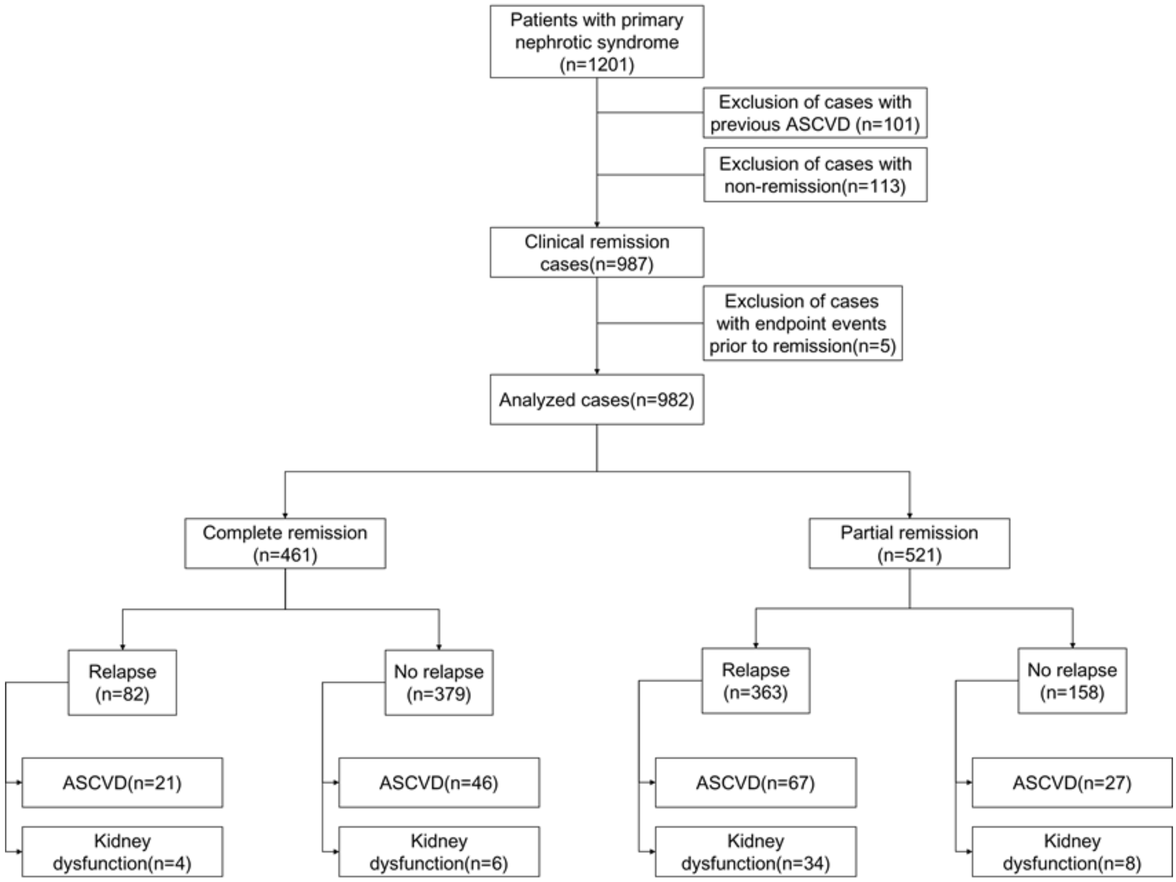

Supplemental Figure 1. Flowchart of patient enrollment. ASCVD: arteriosclerotic cardiovascular disease.
